# *Mycobacterium tuberculosis*/*Mycobacterium bovis* triggered different variations in lipid composition of Bovine Alveolar Macrophages

**DOI:** 10.1038/s41598-022-17531-2

**Published:** 2022-07-30

**Authors:** Yuqi Chen, Huiya Ma, Yangbo Duan, Xueyan Ma, Lihui Tan, Jianjian Dong, Chenkai Jin, Rong Wei

**Affiliations:** 1 Department of Rheumatology and Immunology, The People’s Hospital of Suzhou New District, Suzhou, 215000 China; 2 College of Chemistry and Pharmacy, Northwest A&F University, No.22 Xinong Road, Yangling, 712100 Shaanxi China; 3 State Key Laboratory of Crop Stress Biology for Arid Areas and College of Plant Protection, Northwest A&F University, Yangling, 712100 Shaanxi China

**Keywords:** Cell biology, Microbiology

## Abstract

The lipid composition performs important functions in interaction between macropha-ge and *Mycobacterium tuberculosis* (MTB)/*Mycobacterium bovis* (MB). Current understanding regarding the lipid responses of bovine alveolar macrophage (BAM) to MTB/MB is quite limited. The present study conducted lipidomics and transcriptome to assess alterations in BAM lipid compositions upon MB and MTB infection. We found that both MTB and MB induced glycerophospholipids accumulation in BAM, and MTB induced more alterations in lipid composition. MTB could affect the contents of various lipids, especially ceramide phosphocholines, polystyrene (PS) (17:0/0:0), testolic acid and testosterone acetate. Meanwhile, MB particularly induced accumulation of 1-alkyl,2-acylglycerophosphoinositols. Both MB and MTB suppressed the contents of palmitoleamide, *N*-ethyl arachidonoyl amine, *N*-(1,1-dimethyl-2-hydroxy-ethyl) arachidonoyll amine, eicosanoyl-EA, and PS (O-18:0/17:0) in BAM. Additionally, transcriptome analysis revealed that only MTB triggered genes involved in immune signaling and lipid related pathways in BAM. And MTB mainly activated genes CXCL2 and CXCL3 relevant to NOD-like receptor, IL-17 and TNF to further induce lipid accumulation in BAM, which in turn promoted the formation of foam cells. Meanwhile, time course RT-qPCR results showed that MTB was recognized by BAM to triggered dramatic immune responses, whereas MB could effectively escape the recognition system of BAM, leading rearrangement of lipid metabolisms in BAM at early infection stage. Altogether, the results of the present study provided evidence for changes in lipid metabolism of MTB/MB attacked BAM and contributed to the detection and treatment of zoonotic tuberculosis.

## Introduction

Tuberculosis (TB) is a notorious disease caused by *Mycobacterium tuberculosis* (MTB) and *Mycobacterium bovis* (MB), which are known to severely affect a wide range of mammalian species, especially humans and cattle. In 2016, there were 10.4 million TB patients worldwide, and 10% of TB cases of human were caused by MTB, other 90% cases were caused by the complex of MTBC which were formed mainly by MB^1^. However, the application of currently available treatment strategies for TB is hampered owing to limited understanding regarding the host responses to MTB/MB and lack/absence of accurate clinical indicators. Importantly, species included in *M. tuberculosis* complex (MTBC) are known to exhibit > 99% genetic similarity, however, both MTB and MB have previously been shown to utilize distinct strategies to infect the host. Therefore, it is important to elucidate different and common responses of the host cells towards MB or MTB infections.

Macrophages are known to serve as important parasitic cells for MTB/MB colonization and development. In addition to this, they also perform important functions in innate immune response to defend/fight against MTB/MB infection^2,3^. Lipids providing energy are known to affect macrophage phenotype and function. Thus, disorder of lipid metabolism in macrophages would lead to a decrease in innate immune response associated with the occurrence of MTBC infection. The pathogenesis of tuberculosis is often accompanied by disorders of lipid metabolism^4^. Importantly, it was previously reported that MTB deployed lipids of macrophages as the main carbon source for survival and colonization, which further resulted in host susceptibility^5^. When attacked by MTB, the receptor CD36 present on the surface of macrophage membrane promoted lipid intake by macrophages, while the expression of lipid effluxion receptor was inhibited, which resulted in lipid accumulation and formation of foamy macrophages (FM)^6^. Furthermore, previous studies provided evidences for relationship between lipid accumulation and formation of foamy macrophages (FM) during the process of MTB infection^7^. Interestingly, tumor necrosis factor‐α (TNF‐α) inhibited cholesterol metabolism and promoted lipid uptake in macrophages, further leading to macrophage foaming^8^. These foam macrophages would further secrete pro‐inflammatory factors and metabolic enzymes to induce inflammation, which would suppress the host immune responses towards MTBC. Thus, elucidation of variations in lipid metabolism of macrophages following MTB and MB infection would contribute towards the prevention of tuberculosis occurrence.

Following engulfment by phagocytes, MTB and MB could trigger signals of intracellular pathogen‐associated molecular patterns (PAMPs)^9^. Additionally, various signals involved in defense responses get activated, including signaling pathways relevant to TNF, NOD‐like receptor, and Toll‐like receptor^10^. Subsequently, these signals could invoke various immune responses, including autophagy, apoptosis, ROS burst, and inflammasome activation, to resist MTB/MB^11,12^. It was previously shown that macrophages could further release TNF‐α to form granulomas, which prevented MTBC growth via activation of TLRs signaling^13^. In addition to the critical role of pro‐inflammatory cytokines (such as IFN‐γ, TNF‐α, IL‐6, and IL23), the presence of chemokines, such as CCL2, CCL3, and CXCL10, was also found to be imperative in defense response against MTB infection^10^. However, in case the pathogenic bacteria are not timely killed, the infection would be reactivated along with the failure of immune system, resulting in foaming of macrophages that is available for pathogen development.

The present study conducted integrated lipid metabolomics and transcriptome analyses on bovine alveolar macrophage (BAM) to investigate the changes in lipid metabolism of BAM, following MB and MTB infection. The study identified that the changes in lipid composition varied in BAM following MB and MTB infection. Additionally, transcriptome analysis showed that the occurrence of tuberculosis was generally associated with weakening of autophagy. Importantly, various genes relevant to signaling pathways, involved in innate immune response and lipid metabolism, were found to be specifically induced by MTB, but not MB. Altogether, the present integration analysis of metabolome and transcriptome of MTB/MB‐attacked BAM elucidated basic strategies that could be utilized for the treatment and detection of tuberculosis.

## Materials and methods

### Bacterial culture strains

The *M. bovis* strain (MB) was drug-sensitive and isolated from the lung tissues of cows with tuberculosis. The *M. bovis* was drug-sensitive strain with identical Mycobacterial Interspersed Repetitive-Unit–Variable Number Tandem-Repeat (MIRU-VNTR) Genotypin. And All animal procedures at Animal and Plant Health Agency (APHA) were approved by the APHA Animal Welfare and Ethical Review Board (AWERB), in accordance with relevant guidelines and regulations, and all authors complied with the ARRIVE guidelines. The drug-sensitive *M. tuberculosis* (MTB) strain was genotyped as MIRU-VNTR and isolated from the patient's sputum cultures at the People’s Hospital of Suzhou New District. Written informed consent was obtained from all patients at the time of admission. The People’s Hospital of Suzhou New District granted Ethical approval to carry out the study within its facilities. Both the strains were cultured to the mid-log phase in the Middlebrook 7H9 medium (Becton Dickinson and Company), as suggested by Gao et al.^14^. The *M. bovis* strain culture medium was further supplemented with 0.5% glycerol. The bacteria were cultured for 8 weeks for the infection experiment in a biosafety level-2 facility at the the People’s Hospital of Suzhou New District (Su Zhou, China). Then, the bacteria were harvested from the medium through centrifugation at 3000 rpm for 10 min following one washing in phosphate-buffered saline. To guarantee the same initial infection dose, the bacteria were resuspended in Dulbecco’s Modified Eagle’s Medium (DMEM) with 10% fetal bovine serum (complete medium) to an OD600 of 1 equivalent to 3 × 10^8^ bacteria/mL.

### Cell collection, culture, and infection

This study was approved by the ethics committee for use and care of animals at the Northwest A&F University. Animal tissue samples were pooled and processed following the protocol guidelines recommended in the OIE Manual for Terrestrial Animals. Primary BAM were collected from healthy male bovine lungs by pulmonary lung lavage via tracheal infusion of physiological saline solution as suggested by Romha et al.^15^. In total, 1 L of saline containing 4% antibiotic–antimycotic agent (100×) (SolarBio Life Science) was infused into the lungs via the bronchial tubes. The alveolar lavage fluid containing BAM was then collected. Approximately 4 × 10^7^ total lung cells were cultured in the AIM VTM Medium (research grade, 12055091, Thermo Fisher) supplemented with 10% fetal bovine serum (FBS) and 4× antibiotics (Penicillin, Streptomycin, and Amphotericin B solution), following γ-irradiation for 16 h at 37 °C under 5% CO_2_ before infection. BAM were infected with *M. bovis* strain or *M. tuberculosis* (MTB) at a multiplicity of infection (MOI) of 10 bacilli per alveolar macrophage. Each group of cells from the treatment groups were infected at MOI 10:1 for 4 h, respectively, at 38 °C under 5% CO_2_ atmosphere. We performed this experiment in triplicates for the uninfected control, MB-infected cells, and MTB-infected cells for 4 h of infection. In total, 9 samples were subjected to lipidomics. All MTB or MB infection experiments were conducted in the biosafety level-2 (bsl-2) laboratory of The People’s Hospital of Suzhou New District.

### Metabolites extraction

The samples of macrophages uninfected and infected by MTB or MB with three replicates at 48 h post infection were collected for the lipidomic analyses. The samples were freeze-dried for 20 h at − 60 °C and vacuum ≤ 5 Pa, after which 10 mg of the sample was collected in EP tube and dissolved in 200 μL water. After 30 s of vortexing, the samples were homogenized at 35 Hz for 4 min and sonicated for 5 min, twice. Subsequently, 480 μL of the extract solution (MTBE: MeOH = 5:1) was added, and the samples were further sonicated for 10 min on ice-water bath. Then, the samples were incubated at − 40 ℃ for 1 h and centrifuged at 3000 rpm for 15 min at 4 ℃. Then, 300 μL of the supernatant was transferred to a fresh tube in the vacuum. The dried samples were re-dissolved in 100 μL of 50% methanol in dichloromethane through sonication. The constitution was then centrifuged at 13,000 rpm for 15 min, and the supernatant was transferred to a glass vial for LC/MS analysis. Finally, the lipidomics of each experimental group with three biological replicates were determined.

### LC–MS/MS analysis

The ExionLC Infinity series UHPLC System (AB Sciex) with the Kinetex C18 column (2.1 × 100 mm, 1.7 μm; Phenomen) was used for LC/MS analyses. The mobile phase A consisted of 40% water and 60% acetonitrile with 10 mmol/L ammonium formate, and the mobile phase B consisted of 10% acetonitrile and 90% isopropanol, with 10 mmol/L ammonium formate. The elution gradient was set to 0–12.0 min, 40–100% B; 12.0–13.5 min, 100% B; 13.5–13.7 min, 100–40% B; 13.7–18.0 min, 40% B. The column temperature was set to 45 ℃. The injection volume was 2 μL (pos) and 6 μL (neg), respectively.

The TripleTOF 5600 Mass Spectrometer was used to acquire the MS/MS spectra on an information-dependent basis (IDA) with the acquisition software (Analyst TF 1.7, AB Sciex) to evaluate the full scan survey MS data. The most intensive 12 precursor ions with intensity > 100 were selected for MS/MS at collision energy (CE) of 45 eV. The ESI source conditions were set as follows: Gas 1 as 60 psi, Gas 2 as 60 psi, Curtain Gas as 30 psi, source temperature as 600℃, declustering potential as 100 V, and ion spray voltage floating (ISVF) as 5000 V or − 3800 V in positive or negative modes, respectively^16^.

### Data preprocessing and annotation

An in-house program was developed using the R software for data analysis. The ‘msconvert’ program was used to convert the raw data files (.wiff format) to the mzXML format. Then, the mzxML files were loaded into Lipid Analyzer for data processing^17^. The CentWave algorithm was applied for peak detection by R package XCMS. The lipid identification was achieved by in-house lipid library against their MS/MS spectrum^18,19^. R package metaboanalyst 3.0 was further used to perform Principle Component (PCA) analysis, and Partial Least Squares Discriminant Analysis (PLS-DA), the pathway enrichment and categorize the differential lipid in each pairwise comparison. The pathways with P value < 0.05 was recognized as differential terms. PLSDA module and PCA were performed on the metabolic profiles with metabolite name, sample name and normalized peak area to interpret cluster separation and obtain variable important in projection (VIP) value of each metabolite. The data were further normalized by unit variance scaling. The threshold Log1.5foldchange < − 1 or > 1 and P value < 0.05 were used to identified the differential metabolites.

### Collection of transcriptomic profiles of macrophage following MTB and MB attacks from GEO data repository

As a publicly available transcriptome database, the Gene Expression Omnibus (GEO) contains series high throughput transcriptome profiles. To investigate the responses of macrophage to MTB and MB infection, 9 transcriptome datasets in the “fastq” form were downloaded, including the transcriptome profiles of macrophages to MTB and MB infections, whereas the transcriptome profiles of normal macrophage were set as control. Each group of profiles contained 3 replicates. The detailed descriptions of each transcriptome profile are shown in Table 1.

**Table 1:** List of transcriptome profiles from the GEO database.

Run	BioSample	Experiment	GEO-accession	Condition
SRR6072076	SAMN07693452	SRX3213613	GSM2792506	MTB-infected
SRR6072114	SAMN07693392	SRX3213643	GSM2792535	MTB-infected
SRR6072136	SAMN07693432	SRX3213664	GSM2792556	MTB-infected
SRR7478850	SAMN09623383	SRX4348292	GSM3259598	Control
SRR7478852	SAMN09623381	SRX4348294	GSM3259600	Control
SRR6072086	SAMN07693442	SRX3213623	GSM2792516	Control
SRR7478851	SAMN09623382	SRX4348293	GSM3259599	MB-infected
SRR7478853	SAMN09623380	SRX4348295	GSM3259601	MB-infected
SRR7478855	SAMN09623373	SRX4348297	GSM3259603	MB-infected

### Transcriptome analysis

The reads in each transcriptome profile in the “fastq” form were aligned against the reference genome of Bos taurus (https://www.ncbi.nlm.nih.gov/assembly/GCF_002263795.1) by the software Tophat 2.1.0. Further assembly and quantification of genes were analyzed by software cufflinks, and the GFF file of Bos taurus was used as the reference. The raw Fragments Per Kilobase of transcript per Million (FPKM) data were further used to calculate the relative expression level. Genes matched the condition (log 2 Fold change > 1; FDR < 0.05) were regarded as the differentially expressed genes. The expressions of top 20 DEGs from both transcriptome profiles were re-detected at 48 h post infection. And the correlation between RNA-seq and RT-qPCR was analyzed to investigate the consistency between both experiment conditions.

### Enrichment and protein–protein interaction network analysis

R packages clusterprofiler was applied to analyze gene ontology (GO) and KEGG pathway enrichment analyses on differential genes^20^. A two-tailed Fisher’s exact test was used to test the significance of each GO term or the pathway. The pathway and GO terms with p < 0.05 were recognized as significant categorization. The candidate differential genes were submitted to the STRING database to construct the protein–protein interaction network. The interaction relationship was considered only between the candidate genes, so that the genes not involved in the network were excluded.

### Time course RT-qPCR analysis of genes in response to MTB/MB infection

The samples of macrophages infected by MTB or MB with three replicates at 6, 12, 24 and 48 h post infection were collected for time course RT-qPCR analyses. Total RNA was extracted from each sample using Animal Total RNA Isolation Kit (Sangon) following the manufacturer’s instructions. An Agilent Bioanalyzer 2100 (Agilent Technologies) was used to ensure RNA integrity by determining the RNA integrity number. After being treated with DNase I (Invitrogen) to remove potential DNA contamination, complementary cDNA was synthesized from RNA samples at 42 °C using an EasyScript cDNA Synthesis Kit (Transgene, China). The resulting cDNA products were used for Quantitative real-time PCR analysis with gene-specific primers (Table S5) with SYBR Green staining. Quantitative data were calculated based on the comparative threshold cycle method. The relative expression of each gene was double-normalized against the expression level of the housekeeping gene ACTIN2 and the value of gene expression measured in the non-infected cells. And the normalized expression value after Z-score transformation were shown in the heatmap to exhibit the relative expression changes of each gene in time course. All experiments were repeated three times independently. And two-tailed t tests were used for all statistical analysis. And the P value < 0.05 was used as threshold to identify the significance between each comparison.

## Results

### MTB‐ and MB‐infection induced significant alterations in lipid metabolism of BAM

**Figure 1:**
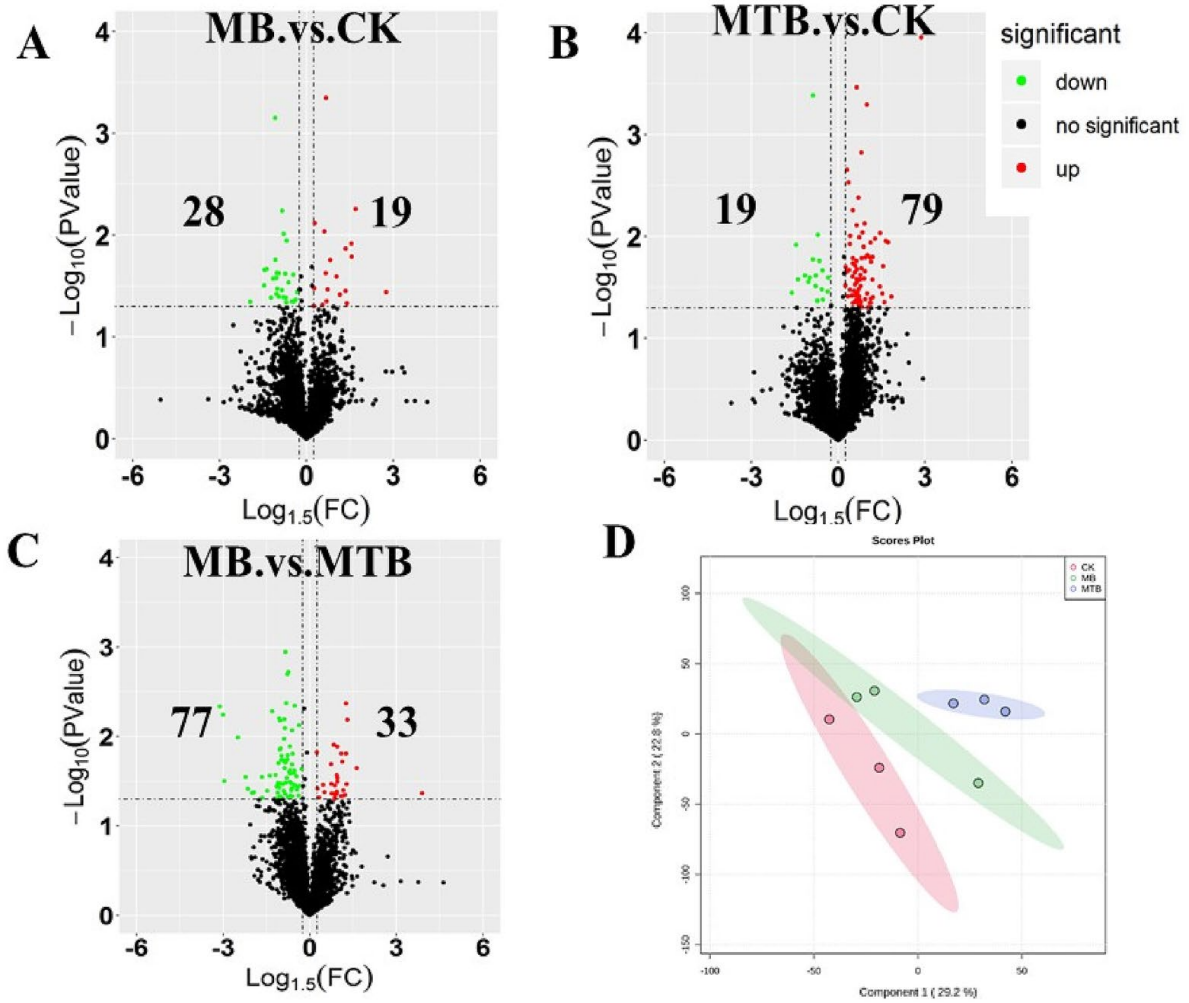
Overview of the alteration of lipid metabolisms of BAM following MTB and MB attacks. (**A**–**C**) Volcano plots of content alterations of lipids in each pairwise comparison (**A**: MB. vs.CK; **B**: MTB. vs. CK; **C**: MB. vs. MTB). The gray points are lipids without significant alteration. The upregulated and downregulated lipids (|Log1.2(Foldchange)|> 1.0; P < 0.05) are labeled by red and green colors, respectively. (**D**) Principal component analysis represents the degree of separation of all samples and the stability between reproductions of each sample groups. Each circle represents a sample, the circles labeled by the same color indicates the samples with the same treatment.

To assess the impact of MB‐ or MTB‐infection on lipid composition of BAM, the present study compared the lipidomics profiles produced by normal BAM and MTB‐ or MB‐infected BAM. It was observed that both pathogenic bacteria (MB and MTB) induced distinct changes in lipid composition of BAM. When compared with normal BAM (CK), 47 differential lipids were produced at 4 h post infection (hpi) with MB, wherein 19 lipids were up‐regulated and 28 lipids were down‐regulated (Fig. 1A, log1.5fc <  − 1 or > 1, P < 0.05). Similarly, 98 differentially expressed metabolites were identified in MTB vs. control group (CK). Importantly, 79 lipids were found to be increased, while 19 lipids were decreased in MTB‐infected BAM (Fig. 1B). For MB vs. MTB comparison, 110 different lipids were identified, 33 of these lipids were up‐regulated, whereas 77 lipids were down‐regulated (Fig. 1C). Following this, the two score plots of the Principle Components Analysis (PCA) models showed a clear separation between each experimental group (Fig. 1D), which indicated that MTB or MB induced significant changes in BAM lipid composition. The top two components, represented by principal component (PC) 1 and PC 2, explained 29.2% and 22.8% of the observed variance/variations, respectively. Since PCA is an unsupervised method and MB‐ and MTB‐infection were not taken into account while defining the components, the patterns shown in the first two PCs were the ones with globally most conspicuous characteristics. These results suggested that both MTB and MB induced significant alterations in BAM lipid composition. Meanwhile, when compared with MB‐infected BAM, more significant differences were induced in BAM lipid composition by MTB‐infection.

### MTB‐ and MB‐infection differentially altered lipid metabolism of BAM

**Figure 2:**
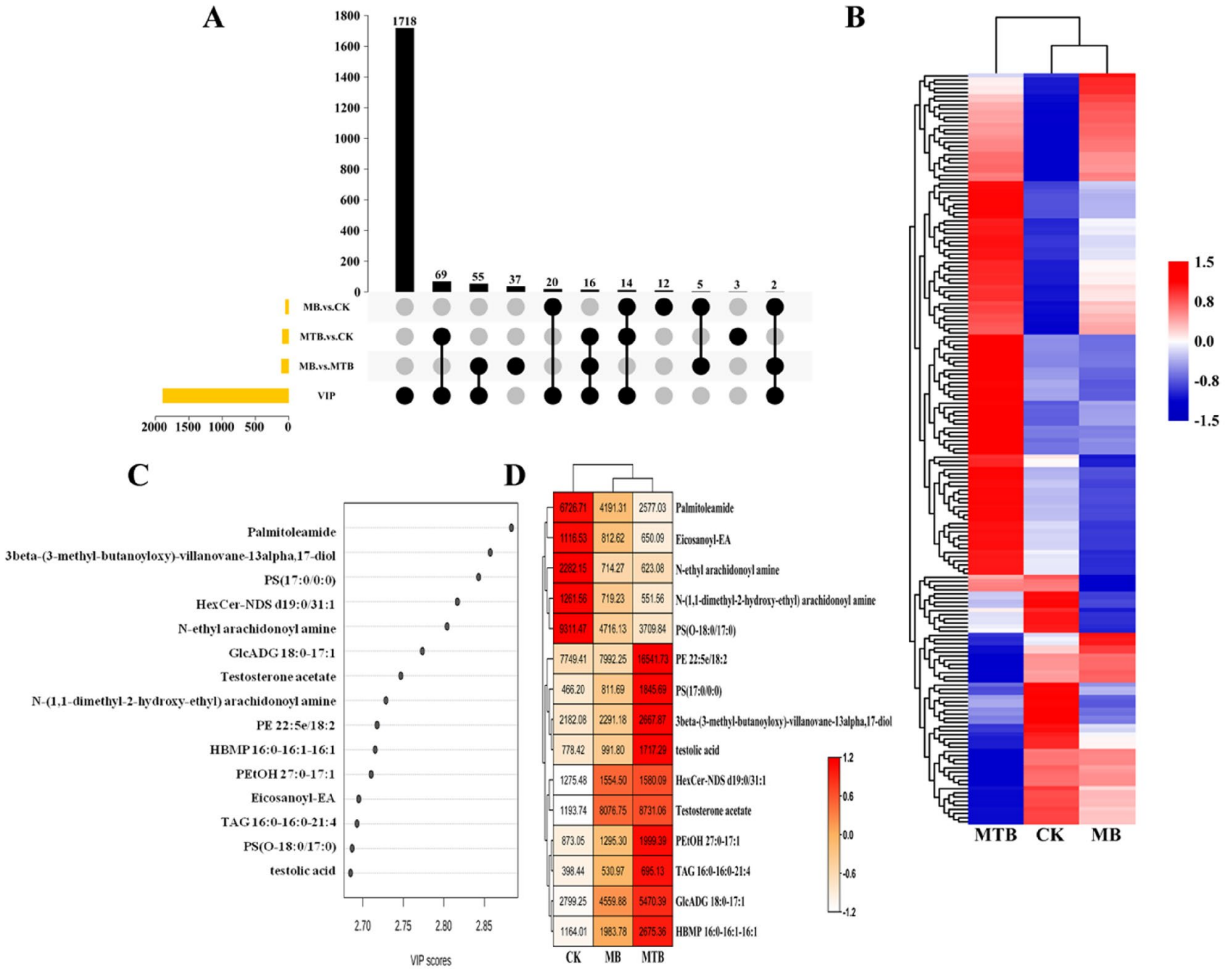
Specific pattern of lipids contents in BAM was induced by MTB- and MB-infection. (**A**) Up-set diagrams representing the overlap of identified differentially expressed lipids in each pairwise comparison. (**B**) Pattern of content variation of differential lipids. The highly increased lipids were shown in red, and the decreased lipids were labeled by green. (**C**) VIP scores of lipids among three experiment treatments. (**D**) Heatmap displaying the level of top 15 lipids (based on VIP score) in each treatment. The scale showed the normalized mean peak area of lipids in each group, and the origin value were shown in cell.

The results for PCA revealed a high correlation of MB‐ and MTB‐infection with specific lipid profile. However, in general, it would be better to consider supervised approaches to specifically focus on relevant differences between MB‐ and MTB‐infection. Therefore, PLS‐DA module was established for better identification of the differences in lipidomics of MB‐ and MTB‐infected BAM, and VIP values were obtained to further assess the significance of each lipid (Fig. 2C). Subsequently, three sets of differentially expressed lipids were identified from MB vs. MTB, MB vs. CK, and MTB vs. CK pairwise comparisons, and the compounds from PLS‐DA module were used to distinguish key compounds (Fig. 2A,D). In particular, seven intersections were obtained, which included 173 lipid compounds, with VIP > 1.5, P < 0.05, and fold change > 1.2. It was observed that these lipids mainly clustered into three modules, based on their relative concentration in each experimental group (Fig. 2B). The contents of most lipids were recorded to be higher in MTB‐infected BAM, whereas the differences in the contents of all these lipids were fairly minor between control and MB‐infected BAM (Fig. 2D).

On the basis of VIP value of PLS‐DA module, it was observed that all the top 15 lipid compounds, which included palmitoleamide, 3β‐(3‐methyl‐butanoyloxy)‐villanovane‐13α,17‐diol, PS (17:0/0:0), HexCer‐NDS d19:0/31:1, *N*‐ethyl arachidonoyl amine, GlcADG 18:0–17:1, testosterone acetate, *N*‐(1,1‐dimethyl‐2‐hydroxy‐ethyl) arachidonoyl amine, PE 22:5e/18:2, and HBMP 16:0‐16:1‐16:1, were dramatically altered in MTB‐ or MB‐infected BAM. However, the levels of these metabolites were not highly increased under MB‐infection (Fig. 2C,D). Among these 15 lipids, the contents of palmitoleamide, *N*‐ethyl arachidonoyl amine, *N*‐(1,1‐dimethyl‐2‐hydroxy‐ethyl) arachidonoyl amine, eicosanoyl‐EA, and PS (O‐18:0/17:0) were found to be commonly suppressed during MTB‐ and MB‐infection (Fig. 2D). Meanwhile, ten lipids, including PE 22:5e/18:2, PS (17:0/0:0), 3β‐(3‐methyl-butanoyloxy)‐villanovane‐13α,17‐diol, testolic acid, HexCer‐NDS d19:0/31:1, testosterone acetate, PEtOH 27:0–17:1, TAG 16:0–16:0–21:4, GlcADG 18:0–17:1, and HBMP 16:0–16:1–16:1 were found to be increased by MTB (Fig. 2D). Typically, no increase in the contents of these 15 lipids was observed in MB‐infected BAM samples (Fig. 2D). All these results reached a credible consensus with PCA plots (Fig. 1A) and indicated that MTB infection induced more significant alterations in BAM lipid composition, as compared to MB‐infection.

### Pathway enrichment analysis on increased metabolites in MTB‐ and MB‐infected BAM

**Figure 3:**
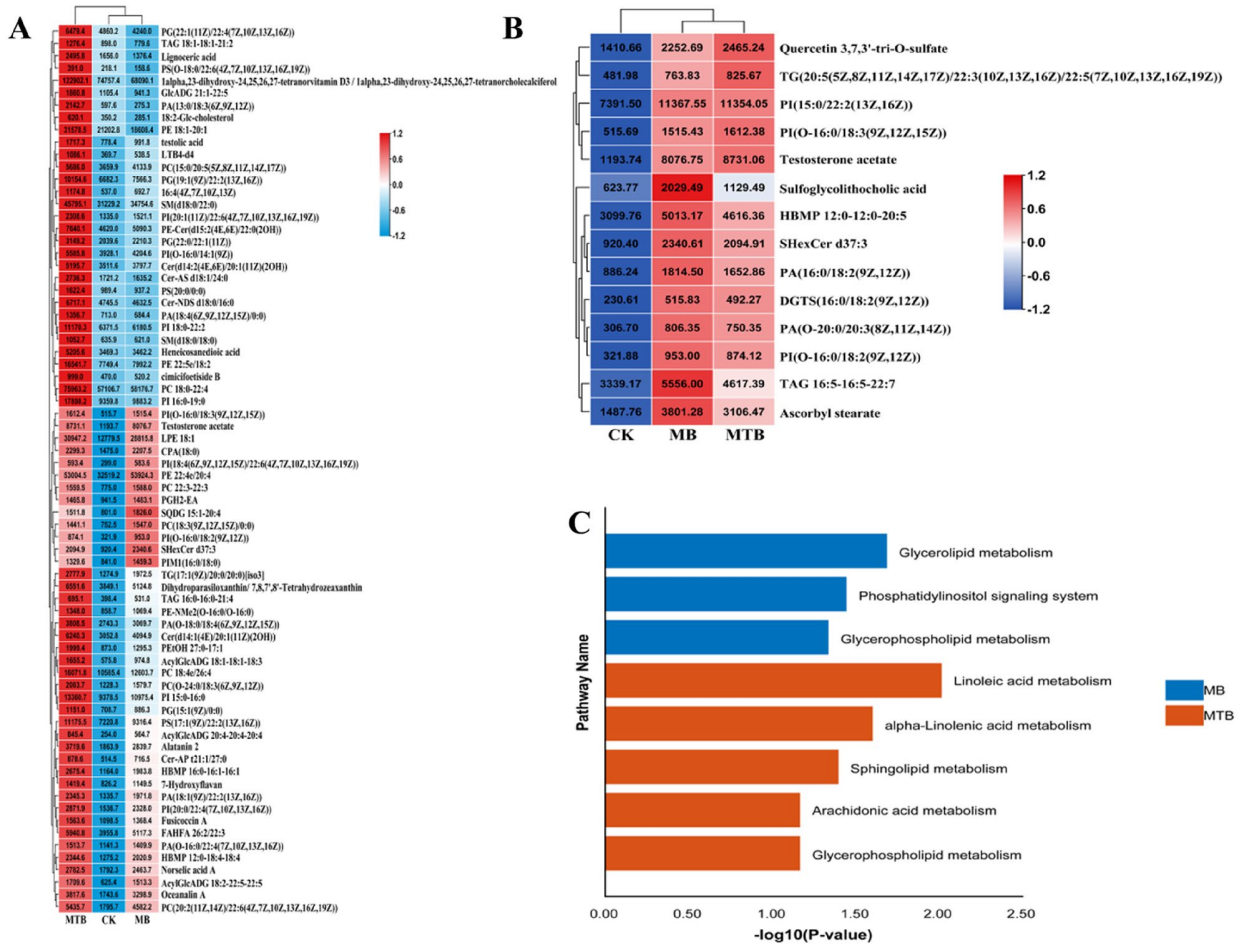
Pathway enrichment analysis on MTB- and MB-induced lipids. (**A**, **B**) Relative abundance of lipids associated with the MTB or MB attacks. Green and red colors indicate metabolites with relative low and high abundance, respectively (**A**: MTB-induced lipids; **B**: MB-induced lipids). (**A**) Pathways associated with MB and MTB infection. Blue represents MB-induced pathways, while red represents MTB-induced ones (P < 0.05).

As shown in Fig. 3, a detailed heatmap clustering was constructed for the lipids with VIP > 1.0, FC > 1.2, and P < 0.05, to clearly analyze the alterations in the contents of these key lipids in MTB‐ and MB‐infected BAM. It was observed that the levels of all these 86 lipids (72 in MTB vs. CK and 14 in MB vs. CK) were higher in MTB‐infected BAM as compared to normal BAM (Fig. 3A,B). Importantly, only 27 of these lipids were found to be highly increased in MB‐infected BAM (Fig. 3A,B).

Subsequently, a pathway enrichment analysis was performed on these two sets of lipids against Kyoto Encyclopedia of Genes and Genomes (KEGG) database. The results showed that glycerolipid metabolism, phosphodylinositol signaling system, and glycerophospholipid metabolism were invoked in MB‐challenged BAM (Fig. 3C). At the same time, MTB infection could induce alterations in linoleic acid metabolism, α‐linolipid metabolism, sphingolipid metabolism, arachidonic acid metabolism, and glycerophospholipid metabolism (Table S1). It was observed that both MTB and MB affected the pattern of glycophospholipid metabolism pathway. It has been previously reported that glycophospholipid metabolism was involved in energy conversion of host^21^. Therefore, it was proposed that MTB and MB could induce an increase in glycophospholipid, which would serve as a nutrient for the development and colonization.

### Classification analysis of MTB‐ and MB‐induced lipids

**Figure 4:**
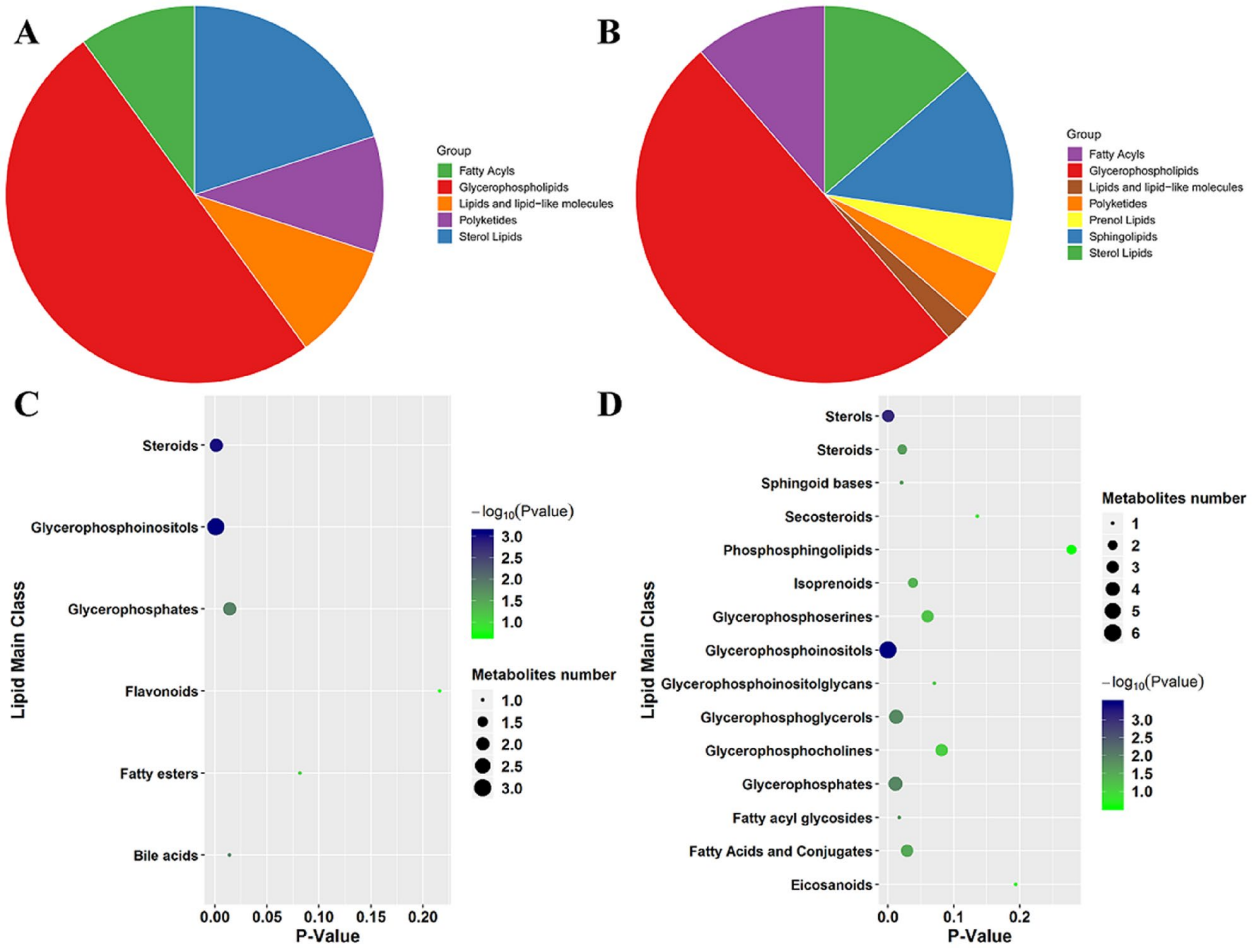
Classification of MTB- and MB-induced lipids. (**A**, **B**) The pie chart represents the main lipid super-classes associated with MTB- (**B**) and MB-infection (**A**). The area represents the percentage of each class. (**C**, **D**) Lipid main class enrichment analysis under MTB (**B**) and MB (**A**) attacks, respectively. The size of circle represents the lipid number in the enriched categories (P < 0.05).

To further explicit the detailed lipid categorizes that were increased in MTB‐infected BAM, a classification enrichment analysis was performed on the compounds accumulated in MTB‐infected BAM. For the categorization based on super‐class level, it was observed that MTB‐infection mainly invoked the accumulation of glycerophospholipids. Importantly, half of the lipids belonged to this category (Fig. 4B). The other lipids were mainly categorized into classes of fatty acids, sterol lipids, and sphingolipids. The lipids relevant to prenol lipids and polyketides class constituted a relatively small percentage of all lipids induced by MTB‐infection (Fig. 4B). For the categorization of main‐class level, it was observed that MTB‐induced lipids relevant to glycerophospholipids mainly belonged to glycerophosphoinositols, glycerophosphates, and glycerophosphoiglycerol classes. At the same time, various other classes were also found to be significantly enriched, which included sterols, fatty acyl glycosides, sphingolipid bases, and steroids classes, with P < 0.05, (Fig. 4D). Various previous studies indicated that sterol‐related lipids represent the dominant category, whose metabolism was dramatically altered by MTB‐infection^7,22^. Further, in terms of sub‐class categorization, it was observed that lipid‐relevant to ceramide phosphocholines, FA glycosides 1‐alkyl,2‐acylgly, *N*‐acylsphingosines, and monoacylglycerol phosphoglycerols were highly enriched in MTB‐induced lipid metabolism network (Table 2).

**Table 2:** Lipid sub-classification of MTB- or MB infection.

	Metabolite set	Metabolites number	P value
MTB infection	1-Alkyl,2-acylglycerophosphoinositols	3	3.22E − 07
	1-Alkyl,2-acylglycerophosphates	2	8.07E − 05
	N-Acylsphingosines	2	9.18E − 05
	C19 steroids	2	2.15E − 04
	Diacylglycerophosphoglycerols	3	0.00111
	Diacylglycerophosphoinositols	3	0.00118
	Ceramide phosphocholines	1	0.00173
	FA glycosides	1	0.00238
	Monoacylglycerophosphoserines	1	0.0067
	Monoacylglycerophosphoglycerols	1	0.0067
	Saturated fatty acids	1	0.00821
	Cycloartanols	1	0.0112
	1-Alkyl,2-acylglycerophosphoserines	1	0.0129
	LPE	1	0.0144
	Sphingoid base analogs	1	0.0157
MB infection	1-Alkyl,2-acylglycerophosphoinositols	2	3.24E − 06
	1-Alkyl,2-acylglycerophosphates	1	0.00272
	Bile acids, alcohols and derivatives	1	0.0029
	C19 steroids	1	0.00444
	C24 bile acids	1	0.00679
	Diacylglycerophosphates	1	0.0398
	Wax monoesters	1	0.0423
	Diacylglycerophosphoinositols	1	0.0424
	Flavones	1	0.126

Subsequently, the study further classified the lipids induced by MB. The results for super‐class level showed that glycerophospholipids still represented the main family that was induced by both MB and MTB (Fig. 4A,B). Therefore, it was speculated that alterations in glycerophospholipids was a common response of macrophages towards MB and MTB infection. Among the main class, lipids belonging to glycerophosphoinositols, glyophosphates, and steroids were the major compounds that were associated with MB‐infection (Fig. 4C). It has been previously reported that sterol‐related lipids could be activated by MB‐infection in BAM^21^. Finally, for sub‐class level, only 1‐alkyl,2‐acylglycerophosphoinositols were found to be highly enriched under MB‐infection (Table 2).

Therefore, the present study revealed that MTB could induce alterations in the contents of ceramide phosphocholines, FA glycosides 1‐alkyl,2‐acylgly, and N‐acylsphingosines, whereas MB induced accumulation of 1‐alkyl,2‐acylglycerophosphoinositols. These lipids might function as specific energy source for the development of both these pathogenic bacteria in vivo, and thus could be used as clinical index/indices for the detection of pathogenic MTB/MB associated with tuberculosis.

### Genes relevant to signaling were dramatically altered by MTB and MB infection

**Figure 5:**
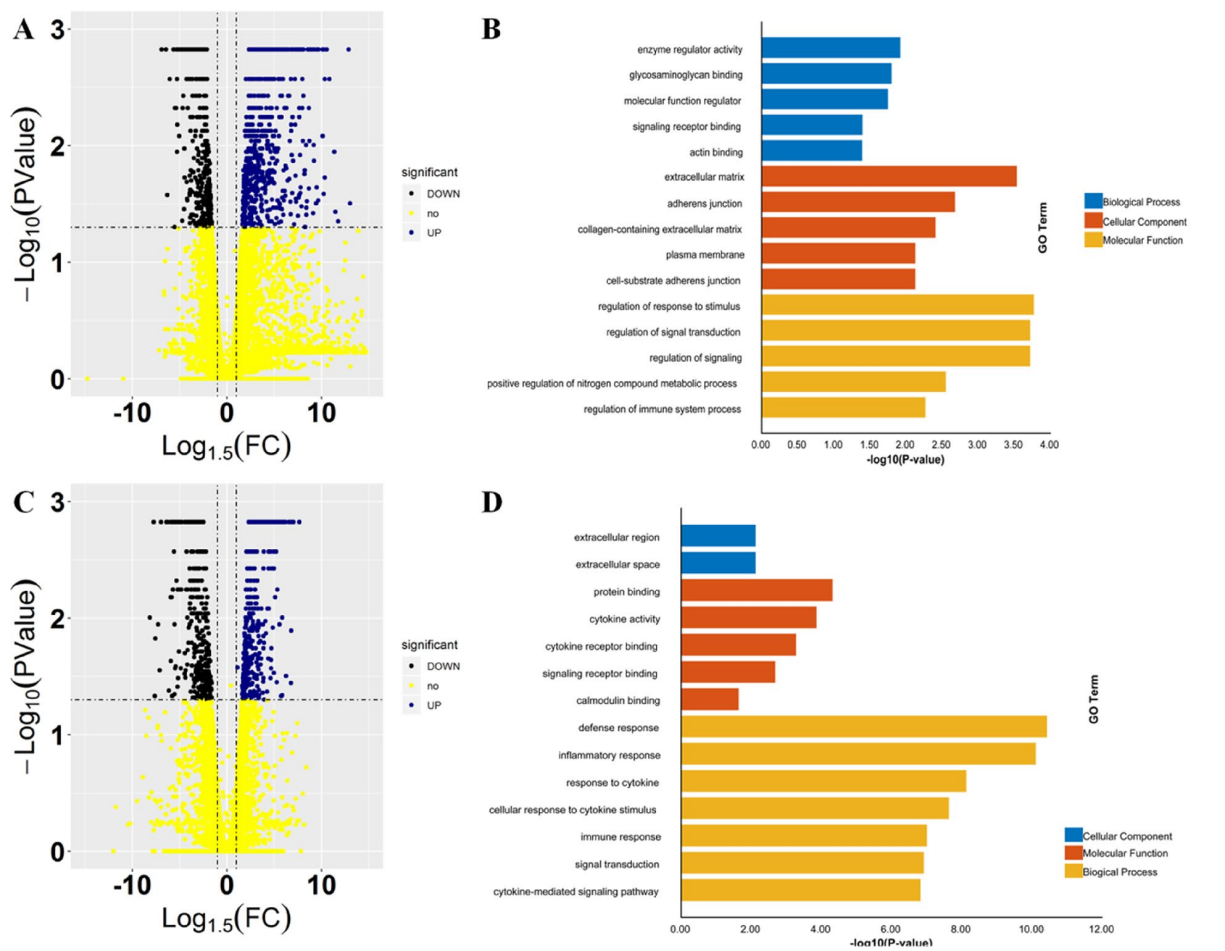
Differentially expressed genes and functional enrichment analysis of BAM infected by MTB and MB. (**A**, **C**) Volcanic map of gene expression in BAM after MB (**A**) and MTB (**B**) infections (Log1.5FC < − 1.0 or > 1.0; q-value < 0.05). (**B**, **D**) All differentially expressed genes infected by MB (**B**) and MTB (**D**) were divided into three GO categories (i.e., cell composition, molecular function, and biological process). Transcriptomic datasets were collected from published articles, as shown in Table 1.

To further elucidate BAM responses to both MTB and MB infection, three sets of transcriptomic profiles with three replicates were obtained from GEO database and analyzed. In particular, transcriptomic data for MTB‐ and MB‐infected BAM were compared with that of normal BAM. For MB vs. CK comparison, 698 and 362 genes were found to be up‐ and down‐regulated, respectively, by MB attacks (Fig. 5A). For MTB vs. CK comparison, 411 genes were up‐regulated and 344 genes were down‐regulated (Fig. 5C). RT-qPCR assay was further deployed to detect the expression of top 20 DEGs from both transcriptome profiles under our experiment condition at 48 h post infection. The results displayed a high correlation between RNA-seq data and RT-qPCR assay, suggesting that the transcriptome data could provide similar results for our study (Supplemental Fig. 1). To clearly understand the function of these genes, GO enrichment analysis was performed on the genes associated with MTB or MB attacks, and the combined functional assignments are provided in Fig. 5. The results of the analyses revealed that the genes that were up‐ and down‐regulated genes under MB‐infection were primarily enriched for “enzyme regulator activity”, “regulation of response to stimulus”, “regulation of signal transduction”, and “regulation of immune system process” (Fig. 5B). This further suggested that MB infection could stimulate the activation of innate immune system of BAM. Furthermore, it was observed that various GO terms relevant to signaling and immune system/response were significantly enriched among the genes associated with MTB‐infection, which included “cytokine activity”, “signaling receptor binding”, “calmodulin binding”, “defense response”, “inflammatory response”, and “immune response” (Fig. 5D). Remarkably, cytokine‐related terms represented the main alterations observed in BAM in response to MTB. Cytokines, such as TNF‐α, IFN‐γ, IL‐6, IL‐12, IL‐23/IL17, and others, are known to be involved in inflammation. In fact, these molecules play important roles in BAM lipid metabolism^10^. Based on these results, it was concluded that MTB induced more dramatic alterations in innate immune response of BAM.

### MTB infection resulted in alterations in lipid‐related metabolisms in BAM

**Figure 6:**
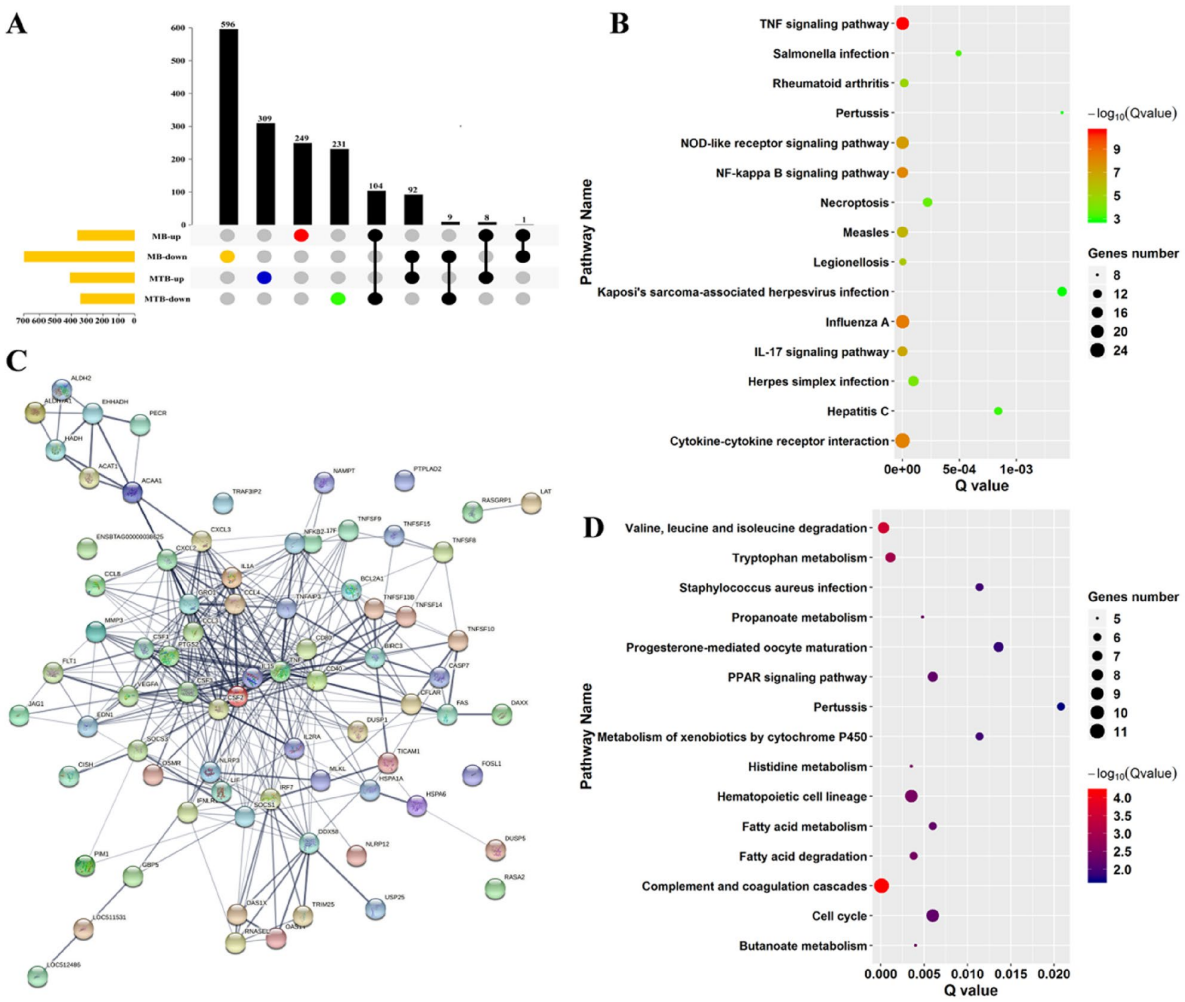
Co-expression network and pathway enrichment analysis of key genes involved in the BAM lipid metabolism. (**A**) Up-set diagrams representing the overlap of identified differentially expressed genes in both MTB- and MB-infected groups. (**B**) Enrichment results of differentially expressed genes associated with MTB-infected BAM. The color shows the significance of pathways. The number of genes involved in the pathway is represented by the dot size. (**C**) Protein–protein interaction network between genes involved in lipid metabolism and signaling pathways. Each circle represented an individual protein. (**D**) Pathway enrichment result of genes associated with MB infection. Transcriptomic datasets were collected from published articles, as shown in Table 1.

To gain detailed insights into the differences in BAM alterations induced by MTB and MB, differentially expressed genes from MB‐ or MTB‐infected and control groups were further annotated by KEGG pathway. The genes affected by MB were mainly enriched in Focal adhesion, Rap1 signaling pathway, MAPK signaling pathway, RAS signaling pathway, and PI3K‐Akt signaling pathway (Table S3). Subsequently, pathway analysis for MTB‐affected genes showed that these genes mainly functioned in signaling pathways relevant to TNF, NOD‐like receptor, NF‐κB, IL‐17, MAPK, RAS, and Toll‐like receptor (Table S3). Both MTB and MB could influence MAPK and RAS signaling pathways, which are important parts of innate immune system. It was further observed that MTB could significantly affect fatty acid‐related pathways, which were not identified in MB vs. CK comparison (Table S3). Thus, series of genes that were only activated or suppressed by MTB infection were obtained and pathway analysis was performed (Fig. 6A). The results showed that various genes involved in lipid metabolism were only suppressed to down‐regulate MTB attacks, whereas the genes involved in immune‐related signaling pathways, such as signaling pathway of TNF, NOD‐like receptor, and NF‐κB, were highly activated in MTB‐challenged BAM (Fig. 6B,D). Moreover, the construction of protein interaction network for these lipid‐ and signaling‐related genes revealed that MTB could result in lipid accumulation in BAM that was mediated via activation of CXCL2 and CXCL3, which were further involved in signaling pathway of NOD‐like receptor, IL‐17, and TNF (Fig. 6C). Altogether, these results provided suitable explanation for the metabolic phenomena involved in MTB infection, wherein MTB‐infection induced the accumulation of various lipid compounds and altered signaling in BAM. Importantly, MB‐infection could not result in such dramatic mechanism.

### MTB triggered immune responses of BAM at early stage

**Figure 7:**
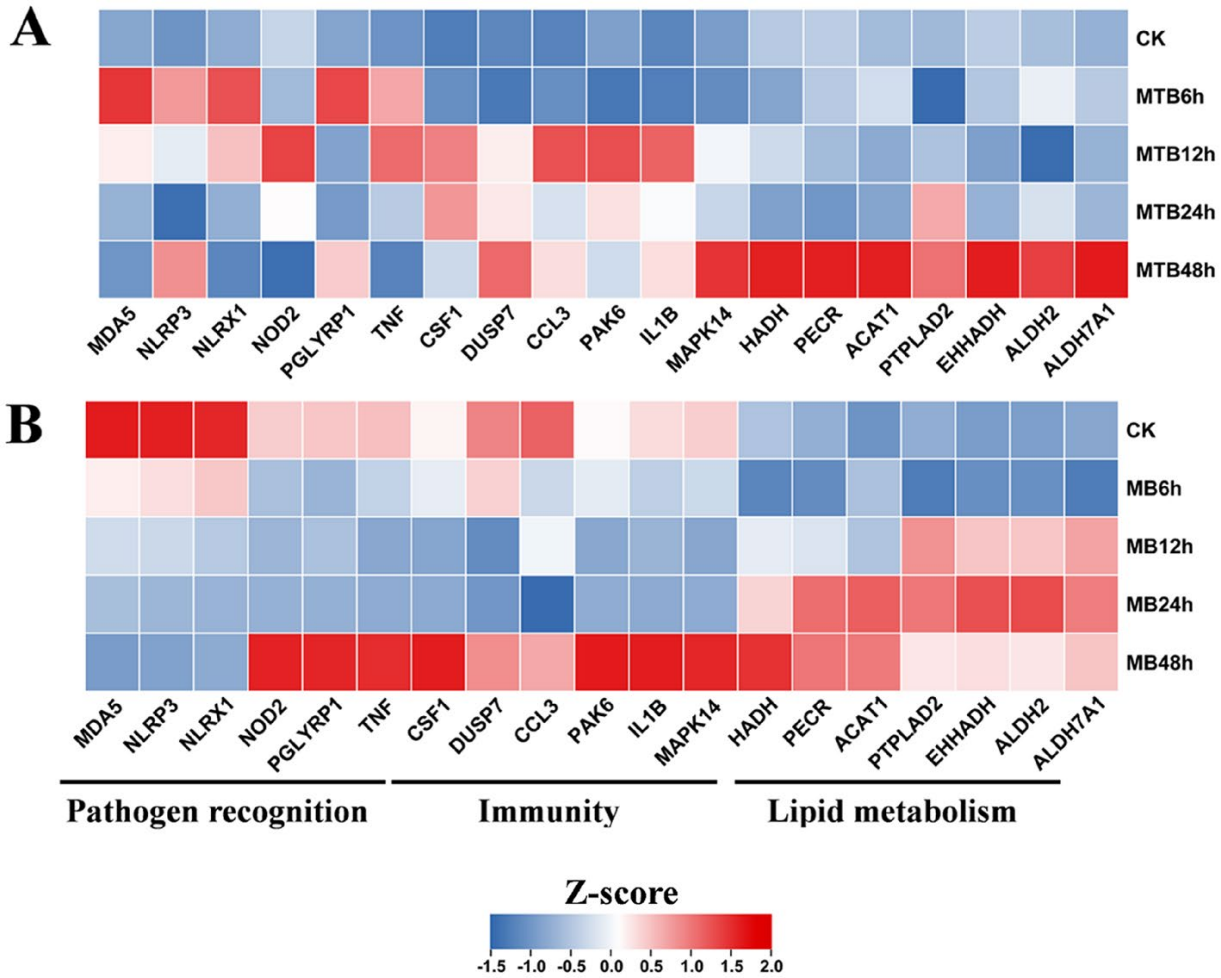
Estimation of the genes relevant to immunity, pathogen recognition and lipid metabolisms in BAM following MTB or MB challenges at time course. (**A**) Heatmap displays the expression levels of genes relevant to immunity, pathogen recognition and lipid metabolisms in MTB-infected BAM at time course. (**B**) The expression levels of representative genes in MB-infected BAM at time course. Scale represents the Z-score of normalized Foldchange value of each genes compared to CK (0 h infection). The up- and down-regulated genes are shown in red and blue, respectively. Two-tailed t tests are used for all statistical analysis, *P* < 0.05.

To investigate the responses of BAM to MB or MTB at different infection stage, a time course RT-qPCR was performed to determined the expression levels of genes relevant to immunity, pathogen recognition and lipid metabolisms (Fig. 7). Expression of numerous genes relevant to pathogen recognition including MDA5, NLRP3, NLRX1, NOD2, PGLYRP1 and TNF were detected in MTB- or MB-infected BAM at time course^23–25^. The results showed that these genes were dramatically triggered to upregulate in BAM at 12 and 24 h post infection with MTB (Fig. 7A). In contrast, some of these genes were only triggered to upregulate at 48 hpi in MB-infected BAM, including NOD2, PGLYRP1 and TNF, and most genes were suppressed to downregulate in BAM by MB at early stage (Fig. 7B). Additionally, genes relevant to immunity were upregulated in BAM following MTB infection at 12 hpi, and were also highly expressed at 24 and 48 hpi (Fig. 7A). However, these immune-related were suppressed by MB infection, and only upregulated at 48 hpi (Fig. 7B). These results suggested that MB could effectively escape the BAM recognition system, whereas MTB were recognized by BAM at early stage and triggered violent immunity responses of BAM to eliminate MTB. For genes relevant to lipid metabolisms, transcriptome results showed the activation of lipid metabolisms in BAM at 48 hpi (Fig. 6). Consensus, our time course RT-qPCR revealed that related genes involved in lipid metabolisms were also only upregulated in BAM at 48 hpi (Fig. 7A). However, MB could triggered upregulated of these genes in BAM at 12, 24 and 48 hpi, suggesting that MB could more effectively alter the composition of lipids in BAM for its colonization (Fig. 7B). Overall, our results showed that MTB could be recognized by BAM to triggered dramatic immune responses, whereas MB could effectively escape the recognition system of BAM, leading rearrangement of lipid metabolisms in BAM.

## Discussion

The main characteristics of MTB and MB pathogenicity primarily involved MTB and MB induced alterations in lipid metabolism to obtain nutrients for their development and colonization in macrophages. In response to this, macrophages could be polarized to M1 type to resist pathogen infection, mediated via activation of lipopolysaccharide (LPS) and Toll‐like receptor (TLR) signaling^26^. In the present study, it was observed that MTB induced more alterations in BAM lipid composition as compared to MB infection. Various lipids, especially glycerophospholipids, were found to be increased in BAM following MTB and MB infection, and thus it was concluded that glycerophospholipids could serve as clinical indicators for tuberculosis detection. Importantly, MTB also induced alterations in the contents of ceramide phosphocholines, FA glycosides 1‐alkyl,2‐acylgly, and *N*‐acylsphingosines, whereas MB only induced accumulation of 1‐alkyl,2‐acylglycerophosphoinositols. Furthermore, MTB dramatically invoked the expression of genes involved in various immune signaling‐ and lipid‐related pathways, including TNF signaling pathway, NOD‐like receptor signaling pathway, Toll‐like receptor signaling pathway, and fatty acid metabolism. However, MB attacks did not induce dramatic activation of signaling pathways or lipid‐related metabolism like MTB. In consensus with aforementioned metabolomics results, these phenomena from transcriptome suggested that MTB induced more alterations in BAM lipid metabolism. Additionally, MTB could activated the expression of genes relevant to pathogen recognition and immunity at early stage, and lipid metabolisms at late stage. In contrast, MB did not triggered genes relevant to pathogen recognition and immunity, but activated lipid metabolisms at early stage. These suggested that MB could escape host immunity and may deploy lipids for its colonization at early infection stage, whereas MTB were recognized by BAM and eliminated by host immune responses. Therefore, the present study provided in‐depth/deep insights into different mechanisms of defense responses elicited in BAM against MTB and MB, which would contribute towards the detection and treatment of human and cattle tuberculosis.

Glycerophospholipids are known to play an important role in the maintenance of cell membrane stability and signal transduction in host cells^27^. The results of the present study showed that MTB and MB attacks induced an increase in the contents of glycerophospholipids, suggesting that both of these microbes might destroy macrophage membrane structure, while host macrophages could invoke intracellular lipid‐related signal to activate defense responses. Besides this, glycerophospholipids are also known to play important roles in energy conversion, vesicle formation, and transmembrane transport^21^. Thus, glycerophospholipids could serve as a nutrient for the host cell or MTB/MB. Additionally, polykelides, one of the upregulated categories, have been previously shown to exhibit anti‐bacterial and anti‐parasitic functions. Thus, an increase in these compounds would contribute towards macrophage defense against MTB/MB. Sterol lipids are important components of cell membrane structure, which are known to play a positive regulatory role in resisting pathogen infection^28^. Consequently, an increase in these lipids would strengthen the host resistance towards MTB/MB attacks. Importantly, it has been previously reported that the levels of sphingolipids were increased in macrophages during MTB infection. Moreover, sphingolipids have been previously shown to be involved in host immune system, which could further induce inflammatory response in macrophages to strengthen host resistance^29^. It was previously reported that MTB infection primarily affected lipid metabolism of macrophages for successful development and colonization^30^. The present data showed an increase in the content of glycerolipids in MTB/MB‐attacked macrophages. Similarly, it has been previously established that MTB induced accumulation of glycerolipids and liposomes as nutrient, for its development in macrophages^31^. Meanwhile, MTB induced the accumulation of lipids mainly via alteration of glycolytic pathway to ketone body synthesis^32^. Thus, disorder of lipid metabolism in macrophages would contribute towards tuberculosis occurrence^33^. Additionally, MTB could further stimulate the accumulation of tumor necrosis factor‐α (TNF‐α) to suppress cholesterol metabolism in macrophages and increase lipid intake, resulting in the formation of FM^8^. The present study reported dramatic activation of TNF‐α signaling pathway in MTB‐infected macrophages, which provided suitable explanation for the pathogenesis of MTB. Importantly, accumulation of lipids could further inhibit autophagy‐related pathways, resulting in the formation of FM, which weakened the phagocytosis of MTB/MB and inhibited their proliferation^34,35^. The present study identified that increased lipids 1‐alkyl and 2‐acylglycerophosphoinositols, 1‐alkyl and 2‐acylglycerophosphates, C19 steroids, and diacylglycerophosphoinositols provided convenience for MB and MTB colonization in macrophages. In fact, these lipids could be used as clinical indicators for tuberculosis.

The phenomenon that more defense‐related responses were activated during MTB‐attacks implied that MB could more effectively evade the defense responses of bovine macrophages than MTB. Among these defense responses, various signaling‐related pathways, including pathways relevant to NF‐κ B signaling, IL‐17 signaling, MAPK signaling, RAS signaling, and Toll‐like receptor signaling, were activated by both MTB and MB attacks. Importantly, only RAP1, MAPK, RAS, and PI3K‐Akt signaling pathways were activated by MB attacks, which suggested that MB could evade the host immune system by certain strategies. Signaling‐related pathways are known to be important parts of immune system of the host that are involved in defense against pathogen infection^36^. Activation of MAPK signaling pathway is known to be inevitable for host response towards pathogens. In particular, it is important for the activation of immunomodulatory molecules, such as TNF‐α and IL, which have been previously shown to be important for macrophages to defend/fight MTB invasion^30^. The present study also reported activation of NF‐κB, IL‐17, and Toll‐like receptor signaling pathways in MTB‐attacked macrophages. These pathways are known to be closely associated with host defense responses, such as various pro‐inflammatory responses and release of antimicrobial effectors^37^. Additionally, activation of IL‐17 signaling pathway has been identified in MTB‐infected macrophages, and IL‐17 could regulate pro‐inflammatory response and chemokines^10,38^. Moreover, activation of peripheral inflammatory cells could produce microbiocidal substances, such as TNF‐α, to inhibit pathogenic bacteria and protect the host cells^13^. Besides this, it has been previously reported that MTB‐induced IFN‐γ, IL‐6, IL‐12, IL‐23/IL17, and other cytokines that performed important functions in response towards MTB, and promoted inflammation^10^. The activation of chemokines could further accelerate the migration of different cell subsets to MTB infected tissues and promote granulomatous formation to kill MTB^39,40^. The present study identified a bunch of MTB‐induced chemokines in macrophages, which included CCL2, CCL3, CCL5, CCL7, CCL12, CXCL2, CXCL8, and CXCL10. These chemokines were involved in pro‐inflammatory response and inhibited MTB. Importantly, protein interaction network showed that CXCL2 and CXCL3 could regulate lipid metabolisms by directly interacting with lipid‐related genes that were associated with MTB infection. Therefore, the study highlighted the role/involvement of chemokines in the interaction mechanisms of MTB and macrophages.

Altogether, the presented integration analysis of transcriptome and lipid metabolomics on BAM and MTB/MB revealed the involvement of different mechanisms in the interaction between BAM and MTB/MB. The study proved that disorder of lipid metabolism in BAM, which resulted in the FM formation, was associated with the pathogenesis of MTB and MB. Importantly, MB did not activate immune responses at early infection stage compared to MTB which triggered dramatic immune responses in BAM. Meanwhile, BAM could deploy a series of signaling pathways and chemokines to defend/fight against MTB infection, whereas MB could evade only a part of BAM defensive responses and may deploy lipid of BAM as nutrient for its colonization at early infection stage. These results provided deeper insights into different defense responses of BAM towards MTB or MB infections, which would further assist in the development of novel strategies for the treatment and detection of tuberculosis in the future.

## Supplementary Information

Supplementary Figure 1.

Supplementary Table 1.

Supplementary Table 2.

Supplementary Table 3.

Supplementary Table 4.

Supplementary Table 5.

## Data Availability

All data generated or analyzed during this study are included in this published article Table S1 Pathway enrichment results of lipid associated with MTB infection. Table S2 Pathway enrichment results of lipid associated with MB infection. Table S3 Pathway enrichment analysis on genes upregulated in MB vs. CK and MTB vs. CK comparisons. Table S4 Origin data of lipidomics of BAM following MTB and MB infection. Table S5 Sequence of primers used in time course RT-qPCR.
