# Supplementary material for: Mycobacterium tuberculosis/Mycobacterium bovis triggered different variations in lipid composition of Bovine Alveolar Macrophages
Source: Sci Rep. 2022 Jul 30;12:13115. doi: 10.1038/s41598-022-17531-2 (PMC9338951; doi:10.1038/s41598-022-17531-2)
Supplement: Supplementary file 1 — Supplementary Figure 1. [file 41598_2022_17531_MOESM1_ESM.pdf]

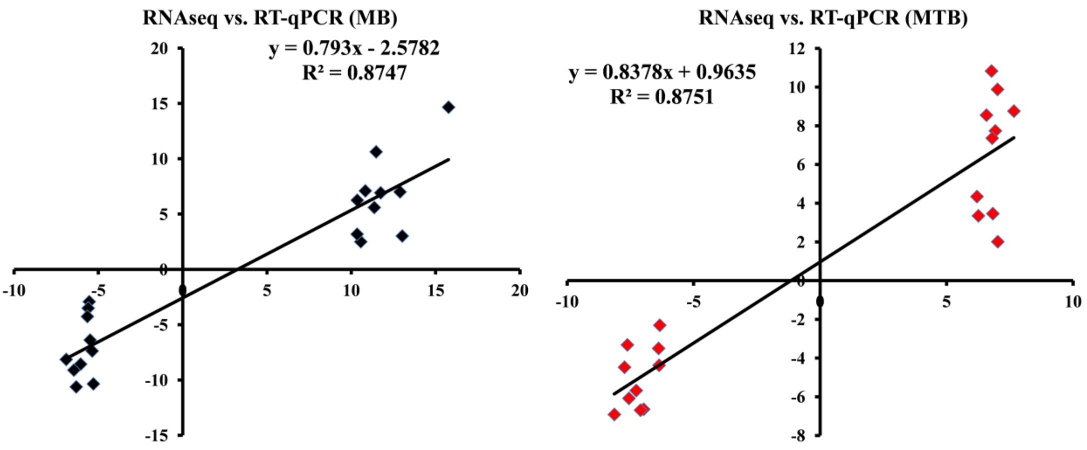

Supplemental Figure 1 Correlation between transcriptome profiles and RT-qPCR data. The top 20 DEGs were re-detected under our experimental conditions using RT-qPCR. Left: correlation analysis between transcriptome data of MB and RT-qPCR assay; Right: MTB.
